# Completeness and selection bias of a Belgian multidisciplinary, registration-based study on the EFFectiveness and quality of Endometrial Cancer Treatment (EFFECT)

**DOI:** 10.1186/s12885-022-09671-5

**Published:** 2022-06-01

**Authors:** Joren Vanbraband, Nancy Van Damme, Gauthier Bouche, Geert Silversmit, Anke De Geyndt, Eric de Jonge, Gerd Jacomen, Frédéric Goffin, Hannelore Denys, Frédéric Amant

**Affiliations:** 1 Biomedical Sciences Group, Department of Oncology, Unit of Gynecological Oncology, KU Leuven, ON4 Herestraat 49, box 1045, 3000 Leuven, Belgium; 2 Belgian Cancer Registry, Koningsstraat 215, box 7, 1210 Brussels, Belgium; 3 The Anticancer Fund, Brusselsesteenweg 11, 1860 Meise, Belgium; 4 Department of Obstetrics and Gynecology, Ziekenhuis Oost-Limburg, Campus Sint-Jan, Schiepse Bos 6, 3600 Genk, Belgium; 5 Laboratory of Pathological Anatomy, AZ Sint-Maarten, Liersesteenweg 435, 2800 Mechelen, Belgium; 6 Department of Obstetrics and Gynecology, CHR de La Citadelle, Boulevard du 12ème de Ligne 1, 4000 Liège, Belgium; 7 Department of Medical Oncology, University Hospital Ghent, Corneel Heymanslaan 10, 9000 Ghent, Belgium; 8 Department of Surgery, Netherlands Cancer Institute, Plesmanlaan 121, 1066CX Amsterdam, The Netherlands

**Keywords:** Corpus uteri cancer, Endometrial cancer, Cancer registration, Completeness, Selection bias, EFFECT

## Abstract

**Background:**

With the aim of obtaining more uniformity and quality in the treatment of corpus uteri cancer in Belgium, the EFFECT project has prospectively collected detailed information on the real-world clinical care offered to 4063 Belgian women with primary corpus uteri cancer. However, as data was collected on a voluntary basis, data may be incomplete and biased. Therefore, this study aimed to assess the completeness and potential selection bias of the EFFECT database.

**Methods:**

Five databases were deterministically coupled by use of the patient’s national social security number. Participation bias was assessed by identifying characteristics associated with hospital participation in EFFECT, if any. Registration bias was assessed by identifying patient, tumor and treatment characteristics associated with patient registration by participating hospitals, if any. Uni- and multivariable logistic regression were applied.

**Results:**

EFFECT covers 56% of all Belgian women diagnosed with primary corpus uteri cancer between 2012 and 2016. These women were registered by 54% of hospitals, which submitted a median of 86% of their patients. Participation of hospitals was found to be biased: low-volume and Walloon-region centers were less likely to participate. Registration of patients by participating hospitals was found to be biased: patients with a less favorable risk profile, with missing data for several clinical-pathological risk factors, that did not undergo curative surgery, and were not discussed in a multidisciplinary tumor board were less likely to be registered.

**Conclusions:**

Due to its voluntary nature, the EFFECT database suffers from a selection bias, both in terms of the hospitals choosing to participate and the patients being included by participating institutions. This study, therefore, highlights the importance of assessing the selection bias that may be present in any study that voluntarily collects clinical data not otherwise routinely collected. Nevertheless, the EFFECT database covers detailed information on the real-world clinical care offered to 56% of all Belgian women diagnosed with corpus uteri cancer between 2012 and 2016, and may therefore act as a powerful tool for measuring and improving the quality of corpus uteri cancer care in Belgium.

**Supplementary Information:**

The online version contains supplementary material available at 10.1186/s12885-022-09671-5.

## Highlights

• This study highlights the feasibility of voluntarily collecting detailed information on the real-world clinical care offered to cancer patients, from diagnosis to follow-up.

• The advantage of this approach is that it enables a more detailed assessment of clinical practice.

• The disadvantage of this approach is that data is at risk of being somewhat incomplete and biased.

**Supplementary Information:**

The online version contains supplementary material available at 10.1186/s12885-022-09671-5.

## Background

Cancer of the uterine corpus is a common disease worldwide, particularly in high- and middle-income countries where the highest incidence rates are seen [1]. In Belgium, with 1352 new cases in 2019, it is the most common cancer of the female genital tract and the fifth most frequent female cancer overall [2]. Furthermore, with 382 related deaths in 2018, it is also the seventh most common cause of cancer-related mortality among Belgian females [3]. This burden is projected to further increase for women over the age of 70 years [4].

In recent years, the management of corpus uteri cancer has changed and improved substantially. However, several aspects of its treatment remain highly controversial [5–8], such as the role of lymphadenectomy in staging and treatment [9, 10]. As a result, wide variations in clinical practice are noticed between hospitals in Belgium, whereby many patients receive a suboptimal quality of care not according to guidelines [11]. In our opinion, this constitutes one of the major concerns for women diagnosed with corpus uteri cancer in Belgium.

The EFFECT (EFFectiveness and quality of Endometrial Cancer Treatment) project was launched with the objective of obtaining more uniformity and quality in the treatment of corpus uteri cancer in Belgium [12]. Quality of care (from diagnosis to follow-up) will be measured by means of quality indicators [13], and improved by means of feedback and benchmarking to the hospitals involved [14]. For this purpose, EFFECT has prospectively collected detailed information on the real-world clinical care offered to 4063 Belgian women diagnosed with primary corpus uteri cancer between 2012 and 2016. This information was collected via an online registration module of the Belgian Cancer Registry, and this on a voluntary basis. The major advantage of this approach is that it enables a highly detailed and meaningful assessment of the clinical care that was offered by hospitals and healthcare teams [15]. However, due to its voluntary nature, the major disadvantage of this approach is that data may potentially be incomplete and suffer from selection bias, as was demonstrated in a highly similar quality of care initiative that was performed in Belgium in the context of rectal cancer (i.e., PROject on CAncer of the REctum; PROCARE) [16, 17]. The presence of such a selection bias is not necessarily problematic, as long as you identify it, characterize it, and take it into account in the analyzation and interpretation of the data. Therefore, this present study aimed to assess and characterize the completeness and potential selection bias of the EFFECT database.

## Methods

### Data sources

Four databases were deterministically coupled by use of the patient’s national social security number as unique identifier: the Belgian Cancer Registry (BCR) database, the database from the InterMutualistic Agency (IMA), the Crossroads Bank for Social Security (CBSS), and the EFFECT database. Due to cancer registration being compulsory in Belgium, the BCR is a national population-based registry that covers basic information (regarding both the patient and the tumor) on at least 98% of all incident cancer diagnoses in Belgium [18, 19]. Consequently, the BCR serves as the gold standard for cancer registration in Belgium. The IMA is a national registry covering information on the (cancer-related) diagnostic and therapeutic procedures, as well as pharmaceuticals, reimbursed to the patient by the Belgian compulsory health insurance. The CBSS covers data on the vital status of the patient, amongst other things. Finally, a fifth database was provided by the public health authorities covering the characteristics of all Belgian hospitals that were recognized as a general acute hospital on December 31^st^, 2016 [20].

### Study population

The following patients were retrieved from the BCR database: all 7239 Belgian women that were diagnosed between 2012 and 2016 with a primary corpus uteri cancer (C54-C55; International Classification of Diseases for Oncology, third edition) eligible for EFFECT. See the online manual for the in- and exclusion criteria of the EFFECT project [21]. Patients for whom IMA data was not available (*n* = 152) were excluded. Furthermore, patients for whom IMA data was less reliable were also excluded: cases with a synchronous malignancy (*n* = 471) or an uncertain incidence date (*n* = 8). Finally, patients for whom the center of main treatment could not be identified were also excluded (*n* = 9) (see below). A synchronous malignancy was defined as a second primary cancer diagnosed in the timeframe of 3 months prior to until 12 months after corpus uteri cancer incidence, regardless of topography and morphology, except non-melanoma skin cancer. This way, a final cohort of 6599 patients was included.

### Hospital allocation and hospital volume

IMA data allowed us to identify the hospital(s) where the patient was treated. By use of the following algorithm, patients were allocated to one specific hospital defined as the center of main treatment: first, if all care was performed in one single hospital, this center was considered as the center of main treatment; second, if care was performed in more than one hospital, the following priority rules were applied for defining the center of main treatment: center of (a) curative surgery, (b) chemotherapy, (c) radiation therapy, (d) hormone therapy, (e) multidisciplinary tumor board (MDT), (f) diagnostic biopsy, and (g) diagnostic imaging. If no treatment centers were known, the patient was assigned to the hospital that registered the patient to the BCR. The center of main treatment could not be identified for nine patients.

A hospital’s volume was then defined as the number of patients that underwent their main treatment in that specific hospital over the period 2012–2016. Volume was categorized in low-, medium-, and high-volume based on the average annual volume and by use of the following cut-off values: < 10, 10–19, and ≥ 20 patients treated on average per year, respectively. Cut-off values are arbitrary and based on expert opinion, as well as on the need to have a balanced repartition of centers and patients over the volume categories.

### Patient subgroups

**Fig. 1:**
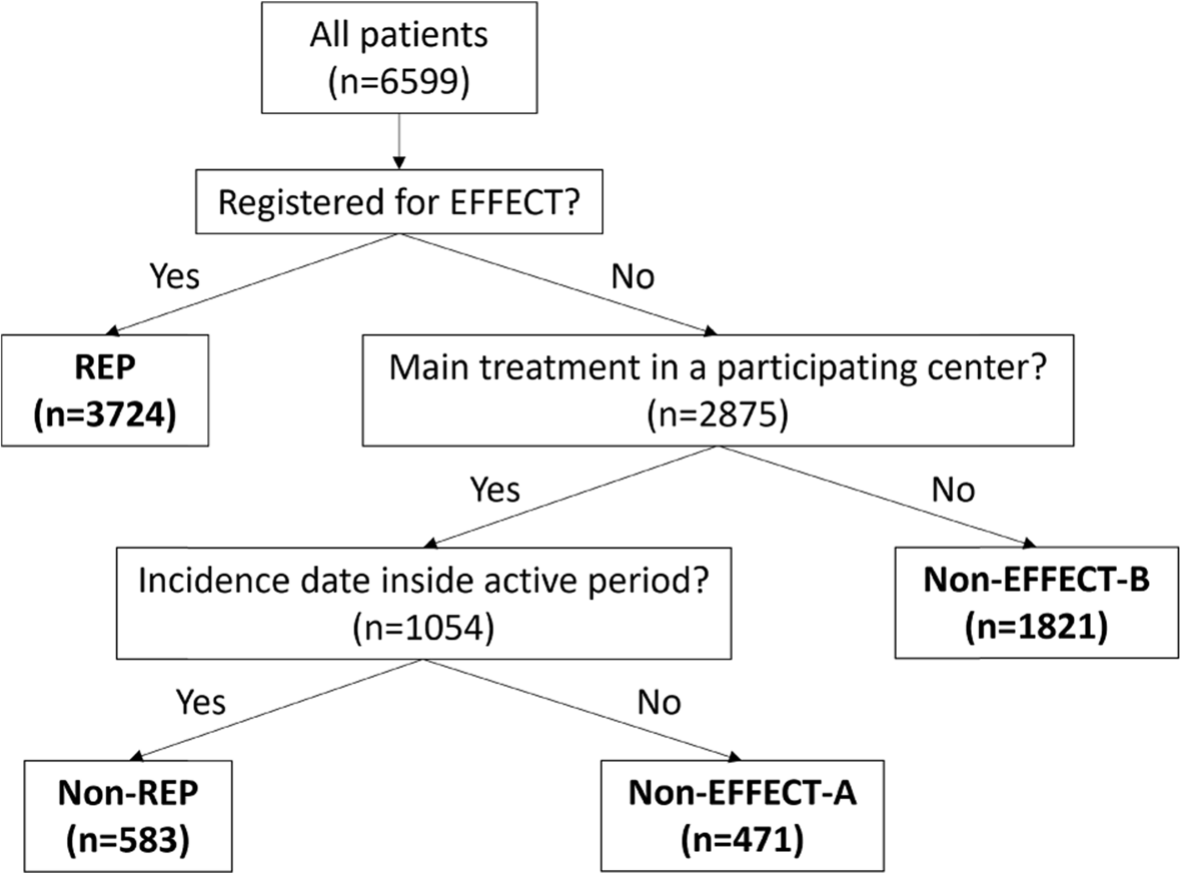
Patient subgroups (flowchart). The objective is to identify those patients that were registered for EFFECT by the participating centers, and those that were not but also should have been. First, based on EFFECT data, the study population was categorized in patients registered and not registered for EFFECT. Next, within the group of non-registered cases, a further distinction was made based on whether main treatment was performed in an EFFECT-participating center and, if yes, whether the patient’s incidence date falls inside the hospital’s active registration period. This way, four patient subgroups were defined: (**a**) patients registered for EFFECT by the participating centers (Registered EFFECT-Patients; REP); (**b**) non-registered patients that underwent their main treatment during a participating center’s active registration period, and therefore also should have been registered (Non-Registered EFFECT-Patients; Non-REP); (**c**) non-registered patients that underwent their main treatment outside of a participating center’s active registration period, and therefore could not have been registered (Non-EFFECT-A); and (d) non-registered patients that underwent their main treatment in a non-participating center, and therefore could not have been registered (Non-EFFECT-B). A participating center’s active registration period was determined by chronologically ranking all its registered cases based on their incidence date, and defined as starting from the first until the last incidence date

The study population was categorized into four patient subgroups: (a) patients registered for EFFECT (Registered EFFECT-Patients, REP); (b) patients not registered for EFFECT that underwent their main treatment during a participating center’s active registration period, and therefore should have been registered (Non-Registered EFFECT-Patients, Non-REP); (c) patients not registered for EFFECT that underwent their main treatment outside of a participating center’s active registration period, and therefore could not have been registered (Non-EFFECT-A); and (d) patients not registered for EFFECT that underwent their main treatment in a non-participating center, and therefore could not have been registered (Non-EFFECT-B). A participating center’s active registration period was determined by chronologically ranking all its registered cases based on their incidence date, and defined as starting from the first until the last incidence date. See Fig. 1 for more detailed information.

### Participation and registration bias

Hospital participation bias was assessed and characterized by identifying characteristics associated with the (non-)participation of hospitals in EFFECT. Patient registration bias was assessed and characterized by identifying patient, tumor and treatment characteristics associated with the (non-)registration of patients by participating hospitals. For the latter, only REP and Non-REP patients were taken into account.

### Statistical analyses

Summary statistics are expressed as medians and (interquartile) ranges for continuous data, and as frequencies and percentages for categorical data. Uni- and multivariable logistic regression were applied for assessing characteristics associated with hospital participation and patient registration in EFFECT. Characteristics to include in the multivariable model were selected based on clinical relevance and results of the univariable analysis (*p* < 0.10 was considered interesting). Goodness-of-fit was assessed by the Hosmer-Lemeshow goodness-of-fit test, the chi-squared test of the Pearson and deviance residuals, and by visual inspection of model residuals. For assessing registration bias, clustering of patients within hospitals (intra-cluster correlations) was taken into account by adding the ‘center of main treatment’ as random effect term to the final model. All statistical tests were two-sided and *p*-values below 0.05 were considered statistically significant. Statistical analyses were performed using SAS 9.4 (SAS Institute, Cary, NC, USA).

## Results

### Descriptives

During the 2012–2016 period, 101 Belgian hospitals were involved in the treatment of corpus uteri cancer, of which 49.5% (*n* = 50) were low-volume, 30.7% (*n* = 31) medium-volume, and 19.8% (*n* = 20) high-volume (Table 1). These hospitals took care of 22.7% (*n* = 1496), 31.9% (*n* = 2106), and 45.4% (*n* = 2997) of cases, respectively (Table 2). Considering the entire study population (*n* = 6599), 60.6% (n = 3998) of cases were diagnosed between the age of 60 years and 79 years. When known, 79.4% (*n* = 4948) of cases were diagnosed with early-stage disease (stage 0-II). Endometrial carcinomas and uterine sarcomas accounted for 95.5% (*n* = 6302) and 4.5% (*n* = 297) of cases, respectively. 64.6% (*n* = 4073) of the carcinomas were of the type I subclass, and 28.8% (*n* = 1812) of the type II subclass. Curative surgery was the primary treatment for 86.9% (*n* = 5732) of patients, with total hysterectomy (TH) being performed most frequently (*n* = 3084; 53.8%). Finally, 92.7% (*n* = 6119) of patients were discussed in at least one MDT meeting (Table 2).

**Table 1:** Center characteristics

	**All centers** **(*n* = 101)**		**Participating** **(*n* = 55)**		**Non-participating (*n* = 46)**		**Participation rate**
**Characteristics**	**N**	**%**	**N**	**%**	**N**	**%**	**%**
**Volume**							
Low (< 10/year)	50	49.5%	17	30.9%	33	71.7%	34.0%
Medium (10–19/year)	31	30.7%	22	40.0%	9	19.6%	71.0%
High (≥ 20/year)	20	19.8%	16	29.1%	4	8.7%	80.0%
**Region**							
Flemish	55	54.5%	35	63.6%	20	43.5%	63.6%
Brussels Capital	10	9.9%	8	14.5%	2	4.3%	80.0%
Walloon	36	35.6%	12	21.8%	24	52.2%	33.3%
**University status**							
General without university character	77	76.2%	39	70.9%	38	82.6%	50.6%
General with university character	17	16.8%	10	18.2%	7	15.2%	58.8%
University hospital	7	6.9%	6	10.9%	1	2.2%	85.7%
**Ownership status**							
Private	73	72.3%	41	74.5%	32	69.6%	56.2%
Public	28	27.7%	14	25.5%	14	30.4%	50.0%

Distribution of volume, region, university status and ownership status for (a) all centers eligible for EFFECT participation (*n* = 101), (b) the participating centers (*n* = 55), and (c) the non-participating centers (*n* = 46). For each subgroup of hospitals with a certain characteristic, participation rate was calculated as the percentage of centers that participated in EFFECT out of all centers

**Table 2:** Patient, tumor and treatment characteristics

	**All patients** **(*n* = 6599)**		**REP** **(*n* = 3724)**^**a**^		**Non-REP** **(*n* = 583)**^**a**^		**Non-Effect-A** **(*n* = 471)**^**a**^		**Non-Effect-B** **(*n* = 1821)**^**a**^	
**Characteristics**	**N**	**%**	**N**	**%**	**N**	**%**	**N**	**%**	**N**	**%**
**Age group**										
< 60 years	1244	18.9%	749	20.1%	111	19.0%	78	16.6%	306	16.8%
60–79 years	3998	60.6%	2273	61.0%	322	55.2%	295	62.6%	1108	60.8%
≥ 80 years	1357	20.6%	702	18.9%	150	25.7%	98	20.8%	407	22.4%
**WHO score**^**b**^										
Known	5733	86.9%	3416	91.7%	450	77.2%	416	88.3%	1451	79.7%
0	1417	24.7%	930	27.2%	70	15.6%	58	13.9%	359	24.7%
1	3988	69.6%	2301	67.4%	337	74.9%	334	80.3%	1016	70.0%
≥ 2	328	5.7%	185	5.4%	43	9.6%	24	5.8%	76	5.2%
Missing	866	13.1%	308	8.3%	133	22.8%	55	11.7%	370	20.3%
**Comorbidity index**^**c**^										
Known	6522	98.8%	3685	99.0%	577	99.0%	465	98.7%	1795	98.6%
0	2318	35.5%	1339	36.3%	213	36.9%	159	34.2%	607	33.8%
1	3000	46.0%	1679	45.6%	269	46.6%	217	46.7%	835	46.5%
≥ 2	1204	18.5%	667	18.1%	95	16.5%	89	19.1%	353	19.7%
Missing	77	1.2%	39	1.0%	6	1.0%	6	1.3%	26	1.4%
**Number of inpatient bed days in year prior to diagnosis**										
0 days	4078	61.8%	2362	63.4%	311	53.3%	289	61.4%	1116	61.3%
1–5 days	1683	25.5%	931	25.0%	172	29.5%	114	24.2%	466	25.6%
6–15 days	480	7.3%	249	6.7%	61	10.5%	41	8.7%	129	7.1%
> 15 days	358	5.4%	182	4.9%	39	6.7%	27	5.7%	110	6.0%
**Multiple tumor status**^**d**^										
No	6267	95.0%	3541	95.1%	541	92.8%	448	95.1%	1737	95.4%
Yes	332	5.0%	183	4.9%	42	7.2%	23	4.9%	84	4.6%
**Center volume**										
Low (< 10/year)	1496	22.7%	520	14.0%	58	9.9%	61	13.0%	857	47.1%
Medium (10–19/year)	2106	31.9%	1210	32.5%	156	26.8%	219	46.5%	521	28.6%
High (≥ 20/year)	2997	45.4%	1994	53.5%	369	63.3%	191	40.6%	443	24.3%
**Incidence year**										
2012	1350	20.5%	684	18.4%	93	16.0%	174	36.9%	399	21.9%
2013	1265	19.2%	717	19.3%	135	23.2%	63	13.4%	350	19.2%
2014	1342	20.3%	848	22.8%	108	18.5%	20	4.2%	366	20.1%
2015	1332	20.2%	788	21.2%	129	22.1%	59	12.5%	356	19.5%
2016	1310	19.9%	687	18.4%	118	20.2%	155	32.9%	350	19.2%
**Combined stage**^**e**^										
Known	6234	94.5%	3615	97.1%	494	84.7%	437	92.8%	1688	92.7%
Stage 0-II	4948	79.4%	2871	79.4%	366	74.1%	355	81.2%	1356	80.3%
Stage III	744	11.9%	455	12.6%	61	12.3%	41	9.4%	187	11.1%
Stage IV	542	8.7%	289	8.0%	67	13.6%	41	9.4%	145	8.6%
Missing (stage X)	365	5.5%	109	2.9%	89	15.3%	34	7.2%	133	7.3%
**Histologic type**^**f**^										
Carcinoma (epithelial)	6302	95.5%	3558	95.5%	528	90.6%	453	96.2%	1763	96.8%
Type I	4073	64.6%	2354	66.2%	318	60.2%	295	65.1%	1106	62.7%
Type II	1812	28.8%	1042	29.3%	149	28.2%	136	30.0%	485	27.5%
Other	417	6.6%	162	4.6%	61	11.6%	22	4.9%	172	9.8%
Sarcoma (mesenchymal)	297	4.5%	166	4.5%	55	9.4%	18	3.8%	58	3.2%
**Differentiation grade**^**g**^										
Known	6181	93.7%	3561	95.6%	522	89.5%	449	95.3%	1649	90.6%
Low-grade (G1-2)	4170	67.5%	2414	67.8%	325	62.3%	304	67.7%	1127	68.3%
High-grade (G3-4)	2011	32.5%	1147	32.2%	197	37.7%	145	32.3%	522	31.7%
Missing	418	6.3%	163	4.4%	61	10.5%	22	4.7%	172	9.4%
**Type of primary treatment**^**h**^										
No treatment	395	6.0%	156	4.2%	73	12.5%	40	8.5%	126	6.9%
Other type	472	7.2%	249	6.7%	69	11.8%	25	5.3%	129	7.1%
Curative surgery	5732	86.9%	3319	89.1%	441	75.6%	406	86.2%	1566	86.0%
Surgery only	3607	62.9%	2125	64.0%	283	64.2%	260	64.0%	939	60.0%
Surgery + (neo)adj. treatment	2125	37.1%	1194	36.0%	158	35.8%	146	36.0%	627	40.0%
**Type of surgery**^**i**^										
No surgery	867	13.1%	405	10.9%	142	24.4%	65	13.8%	255	14.0%
Surgery	5732	86.9%	3319	89.1%	441	75.6%	406	86.2%	1566	86.0%
TH	3084	53.8%	1838	55.4%	245	55.6%	196	48.3%	805	51.4%
TRH	2137	37.3%	1173	35.3%	142	32.2%	163	40.1%	659	42.1%
Debulking	432	7.5%	270	8.1%	44	10.0%	42	10.3%	76	4.9%
Other type	79	1.4%	38	1.1%	10	2.3%	5	1.2%	26	1.7%
**MDT meeting**^**j**^										
No	480	7.3%	125	3.4%	112	19.2%	46	9.8%	197	10.8%
Yes	6119	92.7%	3599	96.6%	471	80.8%	425	90.2%	1624	89.2%
**Biopsy (diagnostic)**										
No	1419	21.5%	780	20.9%	181	31.0%	92	19.5%	366	20.1%
Yes	5180	78.5%	2944	79.1%	402	69.0%	379	80.5%	1455	79.9%
**Imaging (diagnostic)**										
No	68	1.0%	39	1.0%	9	1.5%	3	0.6%	17	0.9%
Yes	6531	99.0%	3685	99.0%	574	98.5%	468	99.4%	1804	99.1%
**30-day post-operative mortality**^**k**^										
Not applicable (NA)	868	13.2%	406	10.9%	142	24.4%	65	13.8%	255	14.0%
Applicable	5731	86.8%	3318	89.1%	441	75.6%	406	86.2%	1566	86.0%
No	5694	99.4%	3306	99.6%	431	97.7%	406	100.0%	1551	99.0%
Yes	37	0.6%	12	0.4%	10	2.3%	0	0.0%	15	1.0%

^a^REP = Registered EFFECT-Patients, Non-REP = Non-Registered EFFECT-Patients, Non-EFFECT-A = non-registered patients treated outside of the active registration period of a participating center, Non-EFFECT-B = non-registered patients treated in a non-participating center

^b^World Health Organization (WHO) performance status score, expressing the patient’s general health condition at diagnosis, ranging from 0 (asymptomatic) to 4 (completely disabled/bedbound) [22]

^c^Index quantifying the prevalence of three major chronic comorbid conditions (i.e., diabetes mellitus, chronic cardiovascular disease, and chronic respiratory disease), ranging from 0 (no comorbidity present) to 3 (all three comorbidities present) [23]

^d^Whether another primary cancer was present in the 5-year period prior to diagnosis, regardless of topography and morphology, except non-melanoma skin cancer

^e^Composite measure of clinical and pathological stage: pathological stage always prevailed over clinical stage, except when clinical stage was IVB (clinical proof of distant metastasis) or pathological stage was missing

^f^Carcinomas were classified in type I (low-grade carcinomas of endometrioid, mucinous or unspecified histology), type II (all high-grade carcinomas, including those of endometrioid, mucinous or unspecified histology), and other carcinomas (of endometrioid, mucinous or unspecified histology and unknown differentiation grade)

^g^Low-grade = well or moderately differentiated (grade 1 or 2), high-grade = poorly or undifferentiated (grade 3 or 4)

^h^Other type = curative or palliative chemo-, radio- and/or hormone therapy

^i^TH = total hysterectomy, TRH = total radical hysterectomy

^j^Multidisciplinary tumor board (MDT)

^k^NA = patients that did not undergo surgery, or were lost to follow-up within the first 30 days post-surgery

### Hospital participation

Of the 101 hospitals treating corpus uteri cancer in the period 2012–2016, 55 (54.5%) did participate in EFFECT. Low-volume centers and centers from the Walloon region were significantly less likely to participate, and are therefore underrepresented in EFFECT. Based on the multivariable model, volume and region were found as the main independent, explanatory factors for the (non-)participation of hospitals in EFFECT (Tables 1 and 3).

**Table 3:** Center characteristics associated with hospital participation in EFFECT

**Center characteristics**	**Univariable regression**				**Multivariable regression**			
	**OR**	**95% CI**	***P*-value (specific)**	***P*-value (overall)**	**OR**	**95% CI**	***P*-value (specific)**	***P*-value (overall)**
**Volume (ref = High)**				< 0.001				< 0.01
Low	0.13	0.04–0.45	< 0.01		0.16	0.04–0.61	< 0.01	
Medium	0.61	0.16–2.34	0.47		0.74	0.17–3.17	0.68	
**Region (ref = Flemish)**				< 0.01				0.04
Brussels Capital	2.29	0.44–11.83	0.32		0.91	0.14–6.00	0.92	
Walloon	0.29	0.12–0.69	< 0.01		0.28	0.10–0.79	0.02	
**University status (ref = University)**				0.12				0.61
Non-university	0.18	0.02–1.57	0.12		0.56	0.06–5.39	0.61	
**Ownership status (ref = Private)**				0.58				0.84
Public	0.78	0.33–1.87	0.58		1.11	0.38–3.24	0.84	

Estimated odds ratios (ORs) for participation of hospitals in EFFECT. ORs are expressed together with their corresponding 95% Wald Confidence Interval (CI) and *P*-value. *P*-value (specific) expresses the statistical significance of the specific comparison with the reference group (ref), whereas *p*-value (overall) expresses the statistical significance of the overall association of the characteristic under investigation with the outcome of interest (i.e., hospital participation status: participating or non-participating)

### Patient registration

**Fig. 2:**
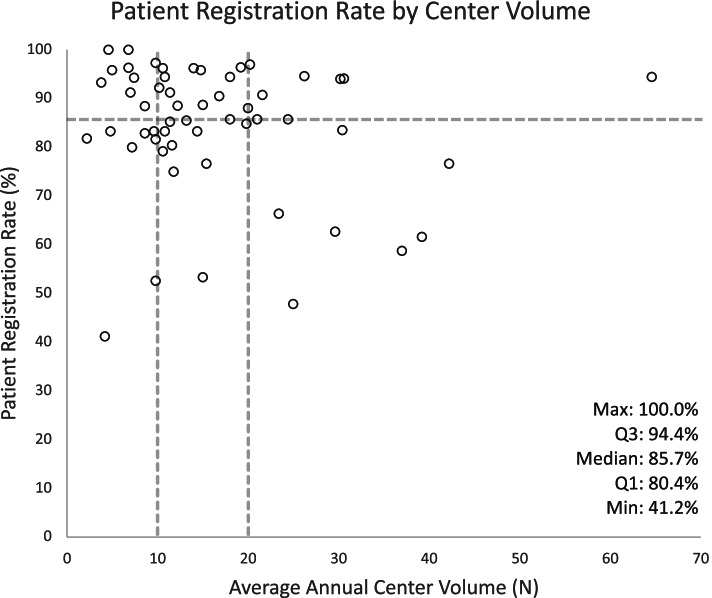
Patient registration rate for the 55 participating centers individually. Each dot represents one individual participating center. The dashed, horizontal line represents the median registration rate (85.7%). The dashed, vertical lines represent the cut-off values applied for making a distinction between low- (< 10 cases/year), medium- (10–19 cases/year), and high-volume (≥ 20 cases/year) centers. A center's registration rate was calculated as the percentage of patients that were registered for EFFECT by that specific hospital (REP) out of the total number of patients that underwent their main treatment during the active registration period of that particular hospital (REP + non-REP). Min = minimum; max = maximum; Q1 = first quartile; Q3 = third quartile

Of the 7239 corpus uteri cancer cases that were retrieved from the BCR database, 4063 (56.1%) were registered in the EFFECT database. Patient registration rate varies widely between the participating centers, which registered a median of 85.7% of cases that were treated during their active registration period (interquartile range = 80.4%-94.4%, range = 41.2%-100.0%) (Fig. 2). Patients aged 80 years and older, with a WHO (World Health Organization) score of ≥ 2 or missing, with a multiple tumor, with stage IV disease or missing stage, and those diagnosed with a uterine sarcoma or other carcinoma (i.e., could not be classified as either a type I or type II carcinoma) were all significantly less likely to be registered for EFFECT by the participating centers. Likewise for patients who did not undergo curative surgery as primary treatment, who were not discussed in an MDT meeting, and who died within the first 30 days post-surgery. Based on the multivariable model; WHO score, combined stage, type of primary treatment, and discussion in an MDT meeting were identified as the main independent, explanatory factors for the (non-)registration of patients by the participating centers (Table 4; Supplementary table 1).

**Table 4:** Patient, tumor and treatment characteristics associated with patient registration in EFFECT

	**Univariable regression**				**Multivariable regression**		
**Characteristics**	**OR**	**95% CI**	***P*-value (specific)**	***P*-value (overall)**	**OR**	**95% CI**	***P*-value (overall)**
**Age group (ref = < 60 years)**				< 0.001			0.40
60–79 years	1.05	0.83–1.32	0.70		0.82	0.62–1.09	
≥ 80 years	0.69	0.53–0.91	< 0.01		0.86	0.60–1.22	
**WHO score (ref = 0)**^**a**^				< 0.0001			< 0.0001
1	0.51	0.39–0.67	< 0.0001		0.49	0.36–0.67	
≥ 2	0.32	0.22–0.49	< 0.0001		0.56	0.34–0.94	
Missing	0.17	0.13–0.24	< 0.0001		0.22	0.15–0.34	
**Comorbidity index (ref = 0)**^**b**^				0.82			0.58
1	0.99	0.82–1.21	0.94		1.00	0.79–1.27	
≥ 2	1.12	0.86–1.45	0.40		1.18	0.86–1.61	
Missing	1.03	0.43–2.47	0.94		1.56	0.55–4.41	
**Number of inpatient bed days in year prior to diagnosis (ref = 0 days)**				< 0.0001			0.13
1–5 days	0.71	0.58–0.87	< 0.01		0.83	0.66–1.05	
6–15 days	0.54	0.40–0.73	< 0.0001		0.77	0.53–1.11	
> 15 days	0.61	0.43–0.89	< 0.01		1.26	0.78–2.03	
**Multiple tumor status (ref = No)**^**c**^				0.02			0.03
Yes	0.67	0.47–0.94	0.02		0.62	0.41–0.95	
**Incidence year (ref = 2012)**				0.03			< 0.001
2013	0.72	0.54–0.96	0.02		0.65	0.47–0.91	
2014	1.07	0.80–1.43	0.66		0.98	0.70–1.39	
2015	0.83	0.62–1.11	0.20		0.63	0.45–0.88	
2016	0.79	0.59–1.06	0.12		0.58	0.41–0.82	
**Combined stage (ref = Stage 0-II)**^**d**^				< 0.0001			< 0.0001
Stage III-IV	0.74	0.60–0.92	< 0.01		0.93	0.69–1.25	
Missing (stage X)	0.16	0.12–0.21	< 0.0001		0.35	0.23–0.53	
**Histologic type (ref = Type I carcinoma)**^**e**^				< 0.0001			0.01
Type II carcinoma	0.95	0.77–1.16	0.59		1.09	0.84–1.41	
Other carcinoma	0.36	0.26–0.49	< 0.0001		0.87	0.57–1.31	
Sarcoma (mesenchymal)	0.41	0.29–0.57	< 0.0001		0.52	0.34–0.79	
**Differentiation grade (ref = Low-grade)**^**f**^				< 0.0001			
High-grade	0.78	0.65–0.95	0.01				
Missing	0.36	0.26–0.49	< 0.0001				
**Type of primary treatment (ref = Surgery + (neo-)adjuvant treatment)**^**g**^				< 0.0001			< 0.0001
No treatment	0.28	0.21–0.39	< 0.0001		0.39	0.25–0.62	
Other type	0.48	0.35–0.65	< 0.0001		0.54	0.37–0.80	
Surgery only	0.99	0.81–1.22	0.95		1.04	0.81–1.35	
**Type of surgery (ref = TH)**^**h, i**^				0.10			
TRH	1.10	0.88–1.37	0.39				
Debulking	0.82	0.58–1.16	0.25				
Other type	0.51	0.25–1.03	0.06				
**MDT meeting (ref = Yes)**^**j**^				< 0.0001			< 0.0001
No	0.15	0.11–0.19	< 0.0001		0.21	0.14–0.31	
**Biopsy (diagnostic; ref = Yes)**				< 0.0001			< 0.01
No	0.59	0.49–0.71	< 0.0001		0.69	0.54–0.88	
**Imaging (diagnostic; ref = Yes)**				0.29			
No	0.67	0.33–1.40	0.29				
**30-day post-operative mortality (ref = No)**^**k**^				< 0.0001			
Yes	0.16	0.07–0.36	< 0.0001				

Estimated odds ratios (ORs) for being registered for EFFECT (REP) when having undergone main treatment during the active registration period of a participating center (REP + Non-REP). ORs are expressed together with their corresponding 95% Wald Confidence Interval (CI) and *p*-value. *P*-value (specific) expresses the statistical significance of the specific comparison with the reference group (ref), whereas *p*-value (overall) expresses the statistical significance of the overall association of the characteristic under investigation with the outcome of interest (i.e., patient registration status: REP or Non-REP)

^a^World Health Organization (WHO) performance status score, expressing the patient’s general health condition at diagnosis, ranging from 0 (asymptomatic) to 4 (completely disabled/ bedbound) [22]

^b^Index quantifying the prevalence of three major chronic comorbid conditions (i.e., diabetes mellitus, chronic cardiovascular disease, and chronic respiratory disease), ranging from 0 (no comorbidity present) to 3 (all three comorbidities present) [23]

^c^Whether another primary cancer was present in the 5-year period prior to diagnosis, regardless of topography and morphology, except non-melanoma skin cancer

^d^Composite measure of clinical and pathological stage: pathological stage always prevailed over clinical stage, except when clinical stage was IVB (clinical proof of distant metastasis) or pathological stage was missing

^e^Carcinomas were classified in type I (low-grade carcinomas of endometrioid, mucinous or unspecified histology), type II (all high-grade carcinomas, including those of endometrioid, mucinous or unspecified histology), and other carcinomas (of endometrioid, mucinous or unspecified histology and unknown differentiation grade)

^f^Low-grade = well or moderately differentiated (grade 1 or 2), high-grade = poorly or undifferentiated (grade 3 or 4)

^g^Other type = curative or palliative chemo-, radio- and/or hormone therapy

^h^TH = total hysterectomy, TRH = total radical hysterectomy

^i^Only patients that underwent surgery were considered

^j^Multidisciplinary tumor board (MDT)

^k^ Only patients that underwent surgery and were not lost to follow-up within the first 30 days post-surgery were considered

Significant differences were also found between patients from participating centers (REP + Non-REP + Non-EFFECT-A) and those from non-participating centers (Non-EFFECT-B). The latter are older (odds ratio (OR)_≥80 years_ = 1.16, 95% confidence interval (CI) = 1.02–1.32), less frequently underwent treatment (OR_treatment_ = 0.80, 95% CI = 0.64–1.00), and were less often discussed at an MDT meeting (OR_MDT_ = 0.52, 95% CI = 0.43–0.63). Surgery rate is not different, but patients from non-participating centers more frequently underwent total radical hysterectomy (TRH) (OR_TRH_ = 1.32, 95% CI = 1.17–1.49) and adjuvant treatment (OR_adj. treat._ = 1.20, 95% CI = 1.06–1.35). Finally, they also more frequently have missing data for WHO score (OR_missing_ = 2.20, 95% CI = 1.90–2.55), combined stage (OR_missing_ = 1.54, 95% CI = 1.24–1.92), and differentiation grade (OR_missing_ = 1.92, 95% CI = 1.57–2.35) (ORs and CIs are calculated based on the data presented in Table 2).

## Discussion

Because of its voluntary nature, this study found the EFFECT database to be incomplete and somewhat biased, both in terms of the hospitals choosing to participate and the patients being registered by participating centers. More precisely, low-volume and Walloon-region centers were less likely to participate in EFFECT. Furthermore, participating hospitals were less likely to include patients with a less favorable risk profile, with missing data for several clinical-pathological risk factors, that did not undergo curative surgery, and that were not discussed in a multidisciplinary tumor board. Finally, clinical practice patterns were found to be different for participating and non-participating institutions.

The observed participation bias could potentially be explained by the following two mechanisms. First, despite our efforts to inform all hospitals about EFFECT, low-volume and Walloon-region centers might have been informed to a lesser extent. Second, particularly low-volume centers might not have disposed of the resources necessary to participate (e.g., time, funding, personnel and technical support). Furthermore, the observed registration bias could potentially be explained by the following three mechanisms. First, in some to many of the participating institutions, EFFECT registration might have been performed by the healthcare team itself, which might have preferred to particularly include patients that they curatively treated. Second, as many aspects of the patient’s treatment scheme were known at the time of first registration, this information might have biased one’s decision whether to include the patient. For instance, when standard of care was offered but refused, one could have decided not to include the patient. Third, EFFECT registration might have been more time-consuming and labor intensive for certain cases. At this point, these mechanisms are merely theoretical and therefore require further investigation.

PROCARE is a quality of care initiative that was performed in Belgium in the context of rectal cancer and was also relying on hospitals to voluntarily register healthcare data [16]. A study by Jegou et al. found the PROCARE database to be incomplete and biased in a highly similar way as EFFECT. More precisely, they also found that low-volume, Walloon-region and non-university centers were less likely to participate. Furthermore, participating centers were less likely to include patients with a less favorable risk profile and who did not undergo surgical resection. This way, the PROCARE database was found to cover 37% of all Belgian rectal cancer patients. These were registered by 72% of centers involved, which included 56% of their cases [17]. Furthermore, a similar underreporting of hospitals and cases has also been described by other clinical audit programs relying on voluntary participation [24–27].

In line with the facilitators and barriers of clinical audit as previously described [28, 29], two survey-based studies by Cornish et al. and Voeten et al. recently found that hospitals and healthcare providers generally think clinical audit programs to be a powerful and relevant tool for improving clinical practice and patient outcomes. However, lack of resources (e.g., technical support, time, personnel and funding) was found to be one of the major reasons for non-participation [30, 31]. Our results reflect these findings, as most hospitals and healthcare teams had a positive attitude towards EFFECT. However, many might not have disposed of the resources necessary to participate, particularly low-volume centers.

Conflicting results have been reported by studies comparing the performance of hospitals and healthcare providers that do participate voluntarily in clinical audit with the performance of those that do not [24, 26, 32, 33]. Similarly, although differences were found in the clinical practice of centers participating and not participating in EFFECT, whether this reflects real differences in quality of care warrants further investigation.

Altogether, for the purpose of measuring and improving the quality of cancer care, these findings highlight the feasibility of voluntarily collecting detailed information on the real-world clinical care offered to the patient, from diagnosis to follow-up. Compared to the use of routinely available administrative data, the major advantage of this approach is that it enables a more detailed and meaningful assessment of clinical practice [15]. Nevertheless, in contrast to administrative databases that are highly complete and free of bias, the major disadvantage of this approach is that such clinical databases are at risk of being incomplete and biased, both in terms of the hospitals choosing to participate and the patients being registered by the participating institutions. As a result, hospitals that would arguably benefit most from quality improvement (i.e., low-volume hospitals) tend not to participate [34–37]. Furthermore, assessing the clinical practice of participating hospitals may be complicated substantially by the bias that tends to be present in their registration of patients. Consequently, to enable meaningful interpretation and feedback, this bias should always be characterized and taken into account. Furthermore, for clinical audit programs to promote quality improvement on the national level, measures should be taken to prevent such selection bias as much as possible, as this requires coverage of all hospitals and patients involved.

Based on the aforementioned mechanisms that could be driving the observed selection bias, we present a couple of methods to potentially reduce the risk of bias in the registration of data, as this would further enhance the potential of clinical audit programs to promote quality improvement. We first suggest to make participation in clinical audit less resource intensive, so that centers and healthcare providers with less resources may also be able to participate. This could potentially be done by making the data extraction and registration process more automated or by giving technical and/or financial support to participating institutions [28–31]. Second, we suggest to ensure that all centers and healthcare teams involved are sufficiently informed about the project. This could possibly be achieved by presenting in person the rationale and importance of the project, which should preferably be done by a colleague renowned in the field [14]. Third, we suggest patient registration to be performed by someone independent from the healthcare team, preferably a data manager specifically trained in cancer registration. Fourth, we suggest the patient to be registered at time of diagnosis, not when many aspects of the treatment scheme are already known. Finally, we suggest rewarding institutions and healthcare teams for their active participation in clinical audit, on the condition that their participation is of sufficient quality (i.e., when a high enough proportion of patients are registered without selection bias). This could potentially be achieved by some sort of accreditation. However, these suggestions are merely theoretical and therefore require further investigation.

The work presented has a couple of limitations that are mainly associated with the databases used. First, although the BCR database has an excellent coverage of nearly all incident cancer cases in Belgium, its data on WHO score, combined stage and differentiation grade was missing for a substantial number of patients. Second, although IMA data was pivotal for this study, it had some major limitations: (a) miscoding or misuse of nomenclature might have occurred; (b) nomenclature was often vague and unspecific, which made detailed analyses and interpretation of data difficult; and (c) the number of patients that underwent a certain medical procedure may have been under- or overestimated due to the impossibility to unambiguously link nomenclature to one specific indication, or to the fact that not all procedures are reimbursed (e.g., when performed in the context of a clinical trial). Different measures were taken to tackle these limitations. For example, cases with missing data were included in the analyses as a separate category within the respective variable, and patients with less reliable IMA data were excluded.

At the same time, these national population-based databases are the major strength of our study: as they are highly complete covering all corpus uteri cancer cases and institutions involved, they allowed us to accurately assess the completeness and potential selection bias of the EFFECT database.

Future studies should focus on unraveling the underlying mechanisms that are driving the selection bias observed in clinical audit programs, as well as on effective ways to counteract these mechanisms. This knowledge could then be applied to further enhance the potential of clinical audit programs to promote quality improvement in healthcare on the national level.

## Conclusion

For the purpose of measuring and improving the quality of cancer care, the present study highlights the feasibility of voluntarily collecting detailed information on the real-world clinical care offered to cancer patients, from diagnosis to follow-up. Compared to the use of routinely available administrative data, the major advantage of this approach is that it enables a more detailed and meaningful assessment of clinical practice. However, in contrast to administrative databases that are highly complete and free of bias, the major disadvantage of this approach is that such clinical databases are at risk of being incomplete and to suffer from selection bias, both in terms of the hospitals choosing to participate and the patients being registered by participating institutions. This bias should therefore always be assessed and characterized, as well as taken into account in the analyzation and interpretation of the data. Furthermore, to really promote quality improvement on the national level, measures should be taken to prevent such bias as much as possible. To conclude, regardless of the observed selection bias, the EFFECT database covers detailed information on the real-world clinical care offered to 56% of all Belgian women diagnosed with corpus uteri cancer between 2012 and 2016. The database may therefore act as a unique and powerful tool for measuring and improving the quality of corpus uteri cancer treatment in Belgium.

## Supplementary Information

**Additional file 1: Supplementary Table 1.** Patient registration rate, overall and specific.

## Data Availability

The data that support the findings and conclusions of this study are presented in the paper, but are not publicly available due to privacy or ethical restrictions. However, data can be made available from the corresponding author upon reasonable request. More concretely, pseudonymized data can be provided within the secured environment of the Belgian Cancer Registry after having been guaranteed that applicable GDPR regulations are taken into account.

## Abbreviations

EFFECT: EFFectiveness and quality of Endometrial Cancer Treatment
PROCARE: PROject on CAncer of the REctum
BCR: Belgian Cancer Registry
IMA: InterMutualistic Agency
CBSS: Crossroads Bank for Social Security
MDT: Multidisciplinary tumor board
REP: Registered EFFECT-patient
Non-REP: Non-registered EFFECT-patient
WHO: World Health Organization
TH: Total hysterectomy
TRH: Total radical hysterectomy
OR: Odds ratio
CI: Confidence interval
