# Supplementary material for: Completeness and selection bias of a Belgian multidisciplinary, registration-based study on the EFFectiveness and quality of Endometrial Cancer Treatment (EFFECT)
Source: BMC Cancer. 2022 Jun 1;22:600. doi: 10.1186/s12885-022-09671-5 (PMC9161534; doi:10.1186/s12885-022-09671-5)
Supplement: Supplementary file 1 — Additional file 1: Supplementary Table 1. Patient registration rate, overall and specific. [file 12885_2022_9671_MOESM1_ESM.docx]

**Supplementary Table 1: Patient registration rate, overall and specific.**

| **Characteristics** |  |  | **Overall registration rate (%)^a^** | **Specific registration rate (%)^b^** |
| --- | --- | --- | --- | --- |
|  |  |  |  |  |
| **Overall (regardless of characteristics)** | |  | 56.4% | 86.5% |
| **Age group** | <60 years | | 60.2% | 87.1% |
|  | 60-79 years | | 56.9% | 87.6% |
|  | ≥80 years | | 51.7% | 82.4% |
| **WHO score^c^** | Known | | 59.6% | 88.4% |
|  |  | 0 | 65.6% | 93.0% |
|  |  | 1 | 57.7% | 87.2% |
|  |  | ≥2 | 56.4% | 81.1% |
|  | Missing | | 35.6% | 69.8% |
| **Comorbidity index^d^** | Known | | 56.5% | 86.5% |
|  |  | 0 | 57.8% | 86.3% |
|  |  | 1 | 56.0% | 86.2% |
|  |  | ≥2 | 55.4% | 87.5% |
|  | Missing | | 50.6% | 86.7% |
| **Number of inpatient bed days in year prior to diagnosis** | 0 days | | 57.9% | 88.4% |
|  | 1-5 days | | 55.3% | 84.4% |
|  | 6-15 days | | 51.9% | 80.3% |
|  | >15 days | | 50.8% | 82.4% |
| **Multiple tumor status^e^** | No |  | 56.5% | 86.7% |
|  | Yes |  | 55.1% | 81.3% |
| **Incidence year** | 2012 | | 50.7% | 88.0% |
|  | 2013 | | 56.7% | 84.2% |
|  | 2014 | | 63.2% | 88.7% |
|  | 2015 | | 59.2% | 85.9% |
|  | 2016 | | 52.4% | 85.3% |
| **Combined stage^f^** | Known | | 58.0% | 88.0% |
|  |  | Stage 0-II | 58.0% | 88.7% |
|  |  | Stage III | 61.2% | 88.2% |
|  |  | Stage IV | 53.3% | 81.2% |
|  | Missing (stage X) | | 29.9% | 55.1% |
| **Histologic type^g^** | Carcinoma (epithelial) | | 56.5% | 87.1% |
|  |  | Type I | 57.8% | 88.1% |
|  |  | Type II | 57.5% | 87.5% |
|  |  | Other | 38.8% | 72.6% |
|  | Sarcoma (mesenchymal) | | 55.9% | 75.1% |
| **Differentiation grade^h^** | Known | | 57.6% | 87.2% |
|  |  | Low-grade (G1-2) | 57.9% | 88.1% |
|  |  | High-grade (G3-4) | 57.0% | 85.3% |
|  | Missing | | 39.0% | 72.8% |
| **Type of primary treatment^i^** | No treatment | | 39.5% | 68.1% |
|  | Other type | | 52.8% | 78.3% |
|  | Curative surgery | | 57.9% | 88.3% |
|  |  | Surgery only | 58.9% | 88.2% |
|  |  | Surgery + (neo)adjuvant treatment | 56.2% | 88.3% |
| **Type of surgery^j^** | No surgery | | 46.7% | 74.0% |
|  | Surgery | | 57.9% | 88.3% |
|  |  | TH | 59.6% | 88.2% |
|  |  | TRH | 54.9% | 89.2% |
|  |  | Debulking | 62.5% | 86.0% |
|  |  | Other type | 48.1% | 79.2% |
| **MDT meeting^k^** | No |  | 26.0% | 52.7% |
|  | Yes |  | 58.8% | 88.4% |
| **Biopsy (diagnostic)** | No |  | 55.0% | 81.2% |
|  | Yes |  | 56.8% | 88.0% |
| **Imaging (diagnostic)** | No |  | 57.4% | 81.3% |
|  | Yes |  | 56.4% | 86.5% |
| **30-day post-operative mortality^l^** | Not applicable (NA) | | 46.8% | 74.1% |
|  | Applicable | | 57.9% | 88.3% |
|  |  | No | 58.1% | 88.5% |
|  |  | Yes | 32.4% | 54.5% |

^a^Overall percentage of patients registered for EFFECT (REP), taking into account all cases that were retrieved from the BCR-database regardless of whether main treatment was performed in a participating center (REP + Non-REP + Non-EFFECT-A + Non-EFFECT-B). ^b^Percentage of patients registered for EFFECT by the participating centers specifically (REP), only taking into account those patients that actually underwent their main treatment during a participating center’s active registration period (REP + Non-REP). ^c^World Health Organization (WHO) performance status score, expressing the patient’s general health condition at diagnosis, ranging from 0 (asymptomatic) to 4 (completely disabled/ bedbound) (22). ^d^Index quantifying the prevalence of three major chronic comorbid conditions (i.e., diabetes mellitus, chronic cardiovascular disease, and chronic respiratory disease), ranging from 0 (no comorbidity present) to 3 (all three comorbidities present) (23). ^e^Whether another primary cancer was present in the 5-year period prior to diagnosis, regardless of topography and morphology, except non-melanoma skin cancer. ^f^Composite measure of clinical and pathological stage: pathological stage always prevailed over clinical stage, except when clinical stage was IVB (clinical proof of distant metastasis) or pathological stage was missing. ^g^Carcinomas were classified in type I (low-grade carcinomas of endometrioid, mucinous or unspecified histology), type II (all high-grade carcinomas, including those of endometrioid, mucinous or unspecified histology), and other carcinoma (of endometrioid, mucinous or unspecified histology and unknown differentiation grade). ^h^Low-grade = well or moderately differentiated (grade 1 or 2), high-grade = poorly or undifferentiated (grade 3 or 4). ^i^Other type = curative or palliative chemo-, radio- and/or hormone therapy. ^j^TH = total hysterectomy, TRH = total radical hysterectomy. ^k^Multidisciplinary tumor board (MDT). ^l^NA = patients that did not undergo surgery, or were lost to follow-up within the first 30 days post-surgery.
